# Aneuploidy and complex genomic rearrangements in cancer evolution

**DOI:** 10.1038/s43018-023-00711-y

**Published:** 2024-01-29

**Authors:** Toby M. Baker, Sara Waise, Maxime Tarabichi, Peter Van Loo

**Affiliations:** 1 The Francis Crick Institute, London, UK; 2 Department of Genetics, The University of Texas MD Anderson Cancer Center, Houston, Texas, USA; 3 Cancer Sciences Unit, University of Southampton, Southampton, UK; 4 Institute for Interdisciplinary Research (IRIBHM), Université Libre de Bruxelles, Brussels, Belgium; 5 Department of Genomic Medicine, The University of Texas MD Anderson Cancer Center, Houston, Texas, USA

## Abstract

Mutational processes that alter large genomic regions occur frequently in developing tumors. They range from simple copy number gains and losses to the shattering and reassembly of entire chromosomes. These catastrophic events, such as chromothripsis, chromoplexy and the formation of extrachromosomal DNA, affect the expression of many genes and therefore have a substantial impact on the fitness of the cells in which they arise. In this Review, we cover large genomic alterations, the mechanisms that cause them, and their impact on tumor development and evolution.

## Introduction

Genomic alterations are a feature universal to all tumors. Among these, single nucleotide variants (SNVs), multi-nucleotide variants (MNVs) and small insertions and deletions (indels), affect only a few base pairs. Other alterations impact much larger regions and affect multiple genes. They include alterations that change the copy number of entire genomic regions, such as aneuploidy, whole genome duplications, and extrachromosomal DNA (ecDNA). Other forms of large-scale genome alterations include genomic catastrophes that cause extensive rearrangements, such as chromothripsis, chromoplexy and breakage-fusion-bridge cycles.

**Figure 1:**
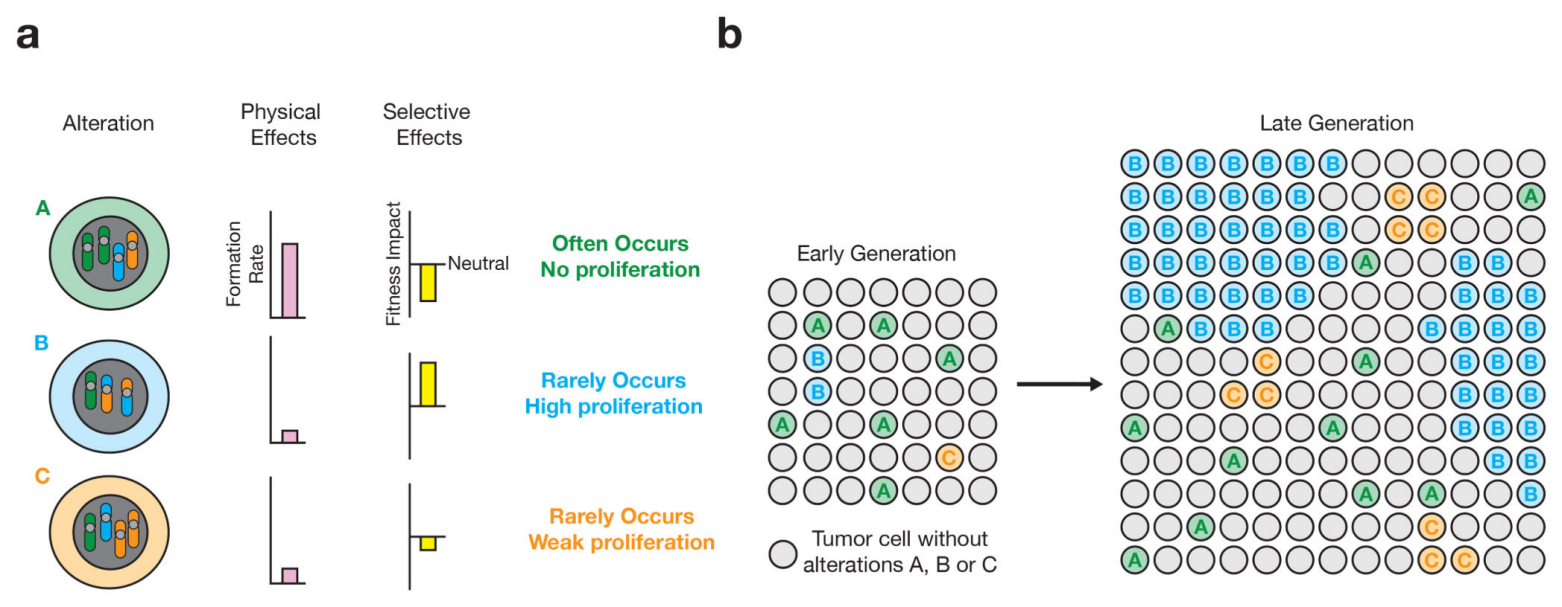
The effect of selective and physical considerations on alteration frequencies. **a**, An illustration of three arbitrary alterations with different rates of formation and selective advantage. **b**, Illustration of the impact of both physical and selective effects on the observed frequencies of large-scale genomic alterations. Alterations with a high formation rate due to physical effects will occur frequently in individual tumor cells, however, a positive selective impact is needed for cells with the alterations to expand relative to non-altered tumor cells. Alteration A will occur more often than other alterations in individual tumor cells, but will not expand due to its reduced fitness. Alterations B and C arise at a similar rate, but B is able to expand much more than C due to the large fitness increase it confers.

The frequency with which genomic alterations are observed depends on a combination of the physical factors that determine their rate of occurrence, and selective factors that determine their rate of fixation in the population, i.e. cell survival and proliferation^1^ (Fig. 1). Even if a particular alteration occurs very frequently in individual cells, it will rarely be observed at the bulk level if it confers a substantial selective disadvantage, as those cells will likely die or fail to proliferate. Conversely, an alteration that occurs very infrequently but confers a substantial selective advantage, may be observed in many tumors. As individual small alterations are numerous and affect only a limited region, both the magnitude of their selective effect and the targets under selection can be reasonably estimated. For example, among thousands of observed individual SNVs in each tumor, only a tiny fraction are thought to be under active selection^2^. In fact, most of the variance in SNV and indel mutation rates along the cancer genome can be explained by local tissue-specific chromatin density and mismatch repair^3,4^. Thus, differences in mutation frequencies along the genome are driven primarily by disparities in rates of occurrence (i.e. physical factors), rather than fitness effects.

Less is known about the fitness impact of large-scale genomic alterations in cancer. Although particular genes are recurrently impacted by large alterations^5^, each individual event typically affects multiple genes simultaneously, and likely has a greater average impact than smaller variants. Therefore, one could hypothesize that the frequencies of large-scale alterations are under substantially larger selection constraints than SNVs and indels. However, as the mutational processes and repair mechanisms associated with large-scale genome alterations are highly complex, physical factors must also have a substantial influence. Here we review large-scale genomic alterations in cancer and how the interplay between physical and selective effects determines their occurrence. We discuss the formation of copy number alterations, whole-genome duplications, and different genomic catastrophes, and their role in tumor initiation and subsequent evolution.

### Aneuploidy

During the late 19^th^ and early 20^th^ centuries, Theodor Boveri and David von Hansemann first noted that aneuploidy, the deviation from a balanced complement of chromosomes in a cell^6–8^, was a feature common to human cancers, leading them to speculate that it may be the root cause of carcinogenesis^9,10^. We now know that cancers also commonly gain and lose smaller genomic regions, as well as whole chromosomes. The term copy number alteration (CNA) describes the complete range of gains and losses occurring in cancer genomes. Aneuploidy and CNAs are caused by chromosomal instability (CIN), a cellular state in which chromosomes, or sections of chromosomes, are gained or lost. As CIN can be episodic^11,12^, an aneuploid state may reflect past rather than ongoing CIN. It is therefore impossible to directly observe CIN from a copy number measurement at a single timepoint, although copy number heterogeneity within a tumor indicates recent CIN. Multi-region^13^ or single-cell sequencing experiments are therefore well suited to assess ongoing CIN. Numerous single-cell sequencing studies have revealed high levels of ongoing CIN in most studied cancer types, including primary tumours^11,14–17^, xenografts^18,19^ and cell lines^20^.

Although CNAs can be any length, they are on aggregate bimodally distributed, as both highly focal events and chromosome-/chromosome arm-level events are frequent. Nevertheless, not all underlying processes lead to bimodality. For example, processes underlying tandem duplications can generate alterations from kilobases to megabases in size^21^.

By encoding features such as CNA size and total copy number across tumors, sets of copy number signatures corresponding to recurrent features of CIN have been identified^22,23^. Many of these signatures have been linked to specific causes of CIN, such as homologous recombination deficiency and chromothripsis, which we review later. Several signatures were also linked to inactivating mutations in particular genes, thereby connecting driver mutations and the initiation of specific CIN processes^22,23^. Associations with response to therapy indicate that classifying CIN has clinical use^23^. However, many signatures have unknown origin, suggesting that multiple processes underlying CIN remain unidentified^22,23^.

Mechanisms that cause CIN and lead to aneuploidy and CNAs have been extensively reviewed elsewhere^24,25^, thus are only briefly summarized here. A major cause of CIN is mitotic defects that cause chromosome mis-segregation during anaphase, leading to daughter cells inheriting an imbalanced set of chromosomes. Mis-attachments of the mitotic spindle to kinetochores are key in generating aneuploidy. Cells ensure that all kinetochores are correctly attached to the mitotic spindle before anaphase through the spindle assembly checkpoint (SAC). Although mutations in SAC genes induce aneuploidy^26^ *in vitro*, they are rare *in vivo* and not thought to be a major contributor to aneuploidy in human cancers^24^. Instead, merotelic attachments, whereby microtubules from both spindle poles attach to a kinetochore^27^ causing lagging chromosomes undetected by the SAC^28^, are a major contributor to chromosome mis-segregation in cancer cell lines with CIN^29^.

Additional centrosomes can also induce CIN. Supernumerary centrosomes can cause a multipolar cell division, resulting in descendants with substantial chromosome mis-segregation. Although multipolar divisions are a source of instability in some cell lines^30^, particularly under conditions of external stress^31^, such catastrophic losses are typically unviable^32^. Instead, additional centrosomes tend to cluster together into two groups such that bipolar division ensues^33,34^. Surplus centromeres primarily drive aneuploidy by causing lagging chromosomes through an increase in merotelic attachments^32^. DNA replication stress has also been linked to increased levels of CIN^35^.

Mutations that inactivate *TP53* have been linked to higher CNA levels in human cancers^36–39^, suggesting that they may induce or allow greater tolerance to CIN. Such mutations are early events in most cancer types^12,40–42^, and may thus initiate some of the earliest CIN in tumor development. However, the cell line evidence for *TP53* inactivation directly causing CIN is mixed, underscoring that the overall context of growth conditions is important^43^. In addition, functional TP53 may more potently inhibit structural chromosomal alterations than chromosome-level gains or losses^44^.

Although gains and losses of all chromosomes are observed in most tumor types^38,45,46^, their frequencies vary greatly. Some aneuploid states are near-ubiquitous in particular cancers, e.g. loss of the 3p arm in clear-cell renal cell carcinomas^47^, 17p loss in ovarian adenocarcinomas^48^, and chromosome 7 gain and chromosome 10 loss in glioblastomas^49^. However, most individual chromosome gains or losses only occur in a minority of tumors.

Some of the differences in CNA frequency across the genome may be due to the physical characteristics of chromosomes. For example, in cells from the female Indian muntjac, a species with very few chromosomes (2n=6), chromosomes with larger kinetochores are more likely to mis-segregate^50^. The DNA content of the centromere on each human chromosome has also been shown to affect mis-segregation^51^. Chromosome distance from the nuclear center at interphase was reported to correlate with mis-segregation likelihood during mitosis^52^. Distance from the nuclear center correlates with chromosome size and therefore, larger chromosomes mis-segregate more frequently^53^. Other physical features that affect CNA frequencies remain to be identified.

Substantial evidence points to selection driving the observed frequencies of different CNAs. The measured gain and loss frequencies of chromosome arms are positively correlated with oncogene and tumor suppressor gene density, respectively^54^. Genes associated with proliferative potential in certain tissues are linked to recurrent CNAs in corresponding cancers^55^. Homozygous inactivation of tumor suppressor genes often occurs through the combination of a loss of one parental allele and an inactivating mutation on the other allele^56^. This could either be a germline mutation conferring familial risk (e.g. *BRCA1* in breast and ovarian cancer^57^), or a somatic mutation (e.g. *TP53*^58^). Furthermore, CNAs that arise independently multiple times over the course of a single tumor’s timeline have been identified, further implying that specific CNAs confer a selective advantage^13,59^. Finally, by considering the breakpoints of telomere- and centromere-bounded copy number changes across thousands of tumors, a recent study posited that selective pressures have a greater impact on the CNA landscape than physical considerations^1^.

Even though certain recurrent CNAs appear actively selected for in different tumor types, the experimental evidence for the tumorigenic and proliferative effects of aneuploidy and ongoing CIN is mixed^6,60–62^. Nevertheless, aneuploidy and CNAs more generally are associated with progression and poorer prognosis in different tumor types^63–66^. Tumors with intermediate CIN are associated with the worst outcomes, with tumors with the most unstable genomes having better prognosis^60,67,68^. Separating the impact of existing CNAs from that of ongoing CIN is challenging, despite high levels of subclonal copy number diversity – likely emerging due to recent CIN – leading to poorer outcomes in multiple cancer types^6,59,69^. Therefore, the effects of CNAs and CIN are highly contextual, as recently reviewed elsewhere^6^.

There is conflicting evidence as to whether CIN helps or hinders tumor immune responses^70,71^, but this may reflect disparate responses to CIN from different components of the immune system. Triple-negative breast cancer cells with CIN were shown to rely on the innate immune system through the cGAS-STING pathway for survival^72^.

CNAs may play a key role in early tumor development. Healthy tissues frequently contain clones with driver mutations linked to tumor development and initiation, yet few CNAs^73–77^, with the exception of the liver and placenta^78,79^. Aneuploidy in pre-malignant lesions has been linked to progression in many cancer types^2^.

Some CNAs appear to be recurrent initiating events in certain cancers. Gains of chromosomes 7, 19 and 20 as well as losses of chromosome 10 are estimated to occur already in childhood in glioblastomas, and potentially even earlier^12^. The loss of chromosome 3p combined with gain of 5q is also a very early event in clear-cell renal cell carcinoma^80^. However, most gains occur over a wide period during tumor evolution, though they follow cancer-type specific trends^12^. Breast and stomach adenocarcinomas accumulate gains throughout their evolution, whereas cancers driven by exogenous mutagens, such as melanomas and lung cancers, typically only accumulate gains after a substantial number of mutations have occurred^12^.

Certain CNAs also appear to be advantageous for later-stage metastasis development, such as loss of chromosome 9p in clear-cell renal cell carcinomas^81^, and gain of 12p in pancreatic adenocarcinomas^82^. Generally, metastases have more aberrant genomes than primary tumors^82–84^, consistent with a late stage of tumor evolution^85,86^. However, this may in part be due to a detection bias: CNAs that are present in the metastasis-seeding cells but at very low overall frequency in the primary tumor are much easier to detect in the metastases due to clonal expansion of the seeding cell(s)^87^. The presence of CIN has been found to increase expression of epithelial-mesenchymal transition genes, necessary for metastatic spread, as well as promoting an inflammatory response that enhances metastasis^83^.

In general, subclonal CNAs appear to be under similar selection pressures as earlier clonal alterations. As with clonal CNAs, the greater the density of oncogenes relative to tumor suppressor genes on a chromosome arm, the more likely it is to have a subclonal gain^13^. The same copy number events have been found to arise independently in different expanding populations within the same tumor, further indicating active selection for some CNAs^13,88^. However, a mathematical model based on multi-region sequencing has suggested that, in colorectal cancer, after an initial burst of CNAs, the accumulation of mutations and CNAs may be under little selective pressure^89^.

Although CNAs continue to accumulate over the course of tumor development, a number of copy number gains appear to occur in periodic bursts. Many clonal copy number gains were shown to arise over a much shorter period than expected by chance, across multiple cancer types^12^. Multiple single-cell and multi-region analyses also point to a burst of high levels of chromosomal instability, followed by lower levels later in tumor evolution^11,14,90^.

### Whole Genome Duplications

Tumor cells can double their entire genome in a single event. Frequency estimates for such whole genome duplications (WGDs) range from 28-37% in pan-cancer cohorts^38,39^ and up to 56% in metastases^84^. However, the indicated higher frequency of WGD in metastases may only be true for certain cancer types^39,91^. WGD rates vary greatly between different cancer types, low in clear-cell renal cell carcinomas, differentiated thyroid cancers, non-Hodgkin lymphomas, and gastrointestinal neuroendocrine tumors, but very high in osteosarcomas and germ-cell tumors^12,39^.

**Figure 2:**
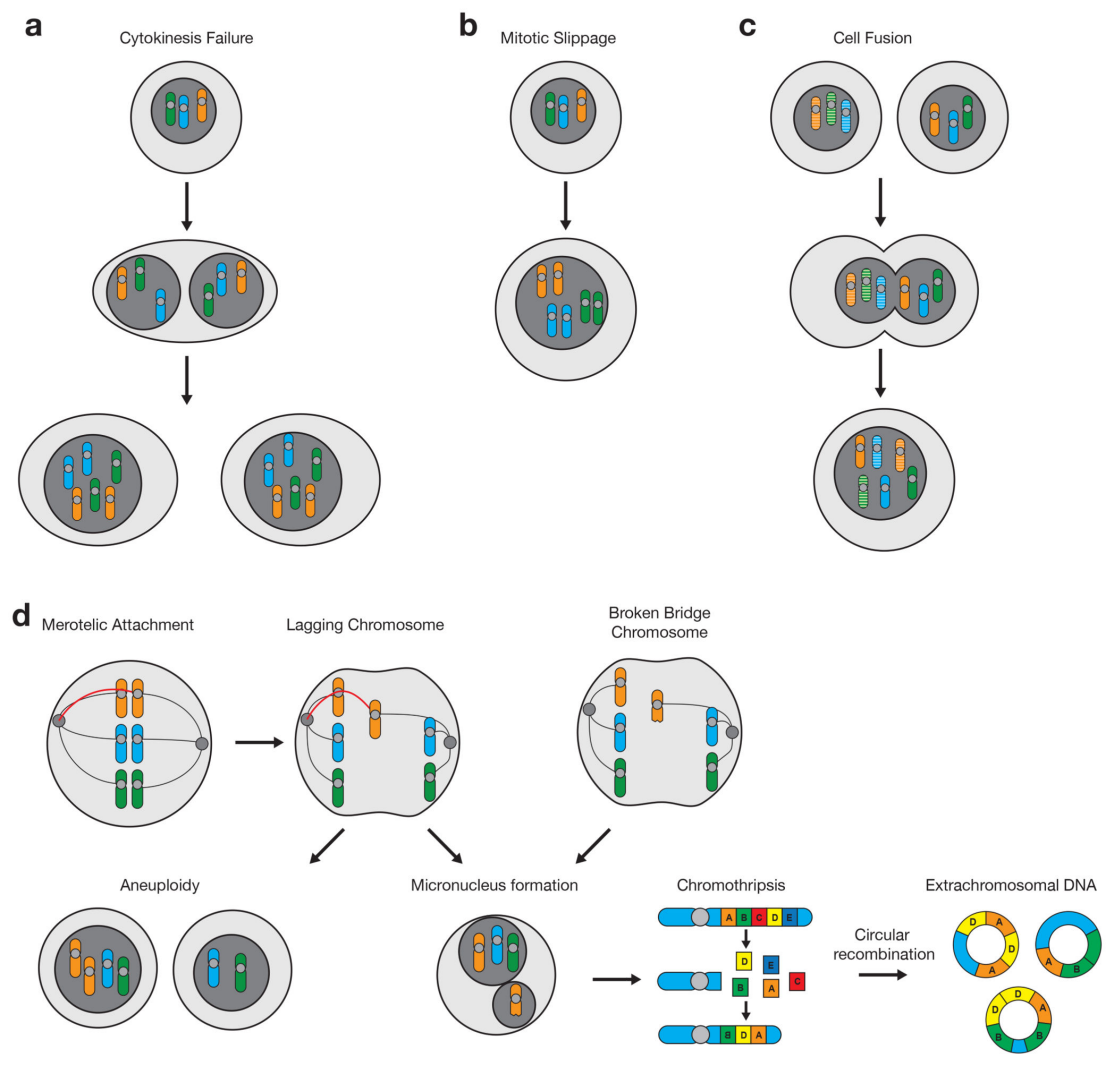
Causes of whole genome duplication and links between complex genomic events. **a**, Cytokinesis failure during mitosis can result in binucleation, and genome duplicated cells in subsequent cell divisions. **b**, Mitotic slippage is a process where mitotic arrest can result in the cell skipping mitosis. **c**, Two tumor cells or a tumor and healthy cell can fuse to form a genome duplicated tumor cell. **d**, Schematic showing how a lagging chromosome event can cause aneuploidy as well as lead to the formation of micronuclei along with broken bridge chromosomes.

WGDs can arise through cytokinesis defects that result in a tetraploid binucleated cell, leading to genome duplicated descendants after a subsequent successful mitosis^92^ (Fig. 2a). Similarly, sustained mitotic arrest by the spindle assembly checkpoint can result in the cell exiting mitosis without completion, or skipping it completely^93^ (Fig. 2b). Among the factors that can cause these cytokinesis defects and mitotic failures are mutations or aberrant expression of well-known tumor suppressor genes such as APC and BRCA2^45,92^, as well as telomere crisis^94^. Cell fusion, promoted by viral infection^95^, can also cause WGDs (Fig. 2c). Cell fusion frequency in tumor development remains unclear, as it is difficult to observe, although it has been identified through the presence of donor DNA in tumors from patients who received bone marrow transplants^96^.

WGD has been linked to increase in CIN and subsequent aneuploidy, for instance by generating additional centrosomes, as discussed earlier. Additionally, a cell that has recently undergone WGD may have insufficient machinery to fully replicate, causing both under- and over-replicated regions in progeny cells^97^. In tumors with a subclonal WGD, subclones with WGD have more CNAs than non-WGD subclones, further supporting an increase in CIN caused by WGD^13^. The relative frequency of whole chromosome aneuploidies compared to arm-level aneuploidies is much higher for WGD than non-WGD tumors, pointing towards mis-segregation as a major consequence of increased instability arising from WGD^98^. WGDs also appear to increase tolerance to aneuploidy^99^. Indeed, the average copy number of each chromosome in genome duplicated tumors is around three, indicating substantial chromosomal loss in duplicated cells^39^.

As WGD doubles the DNA content to be replicated per cell cycle, it imposes a substantial burden on the cell. Therefore, the high frequency of WGD in tumors implies that it may confer a selective advantage. Consistent with this, WGD has been linked to poorer survival in multiple cancer types^39^. Furthermore, *TP53* knock-out cell line experiments indicate that only tetraploid cells give rise to tumors when transplanted into mice^100,101^.

WGD has been proposed to mitigate the effect of deleterious mutations in cancer development, by providing additional copies of essential genes^102^. Mutations in essential genes in tumor genomic regions with haploid loss of heterozygosity are under negative selection^103^, which is relieved following WGD^102^. Before WGD, losses are less frequent on arms with a high concentration of essential genes, but no such relationship is observed for post-WGD losses^102^. WGDs therefore likely change the selection landscape of copy number losses. In contrast, WGDs appear to have little impact on the fitness landscape of copy number gains. Although the frequency of specific gains differs between WGD and non-WGD tumors (such as gains of chromosomes 19 and 20 in glioblastoma, which are considerably more common in non-WGD tumors^104^), in most cases the event frequencies are highly correlated^98,104^.

Another potential advantage of genome duplications is that increased CIN post-WGD provides more opportunities for CNAs with high selective impact and therapy resistance^105,106^. Moreover, WGD events were reported to promote tumorigenesis through changes to chromatin organization^101^. Separately, computational simulations were employed to demonstrate that WGD allows cancer cells to reach an optimal triploid copy number state with lower fitness cost than through sequential independent chromosome gains^107^.

WGDs often appear within the clonal evolution period of cancer^13^, typically following initiating driver mutations and CNAs^12^. Real-time estimates point to WGDs occurring years and sometimes decades before diagnosis^12^. When they both occur in the same tumors, a *TP53* inactivating mutation almost always precedes WGD, supporting that *TP53* mutations either increase WGD occurrence, or increase tolerance to WGD^39,108^.

### Structural variants

Genomic rearrangements, also called structural variants (SVs), are a key source of somatic mutations in human cancer^21,109^. Although some of these SVs are interlinked and share underlying causes with CNAs (Fig. 2d), they form a distinct semantic class of changes. These may be classified as simple SVs, occurring in isolation (*e.g*. translocations and inversions)^110,111^, or as part of more complex events where multiple rearrangements may occur simultaneously, or through a linked series of events^112–114^. Recent whole-genome sequencing studies have enabled identification of novel rearrangement patterns, and refinement of the classification of known processes^21,114,115^, indicating that at least some of these events have a key role in the initiation and progression of cancer^113,116^.

DNA replication and repair mechanisms have key roles in the generation of both SVs and CNAs^117–119^. For example, breakpoints in chromothripsis, ecDNA, and CNA events have been observed to show microhomology^110,120,121^, indicative of breaks repaired by non-homologous end joining (NHEJ, which in contrast to homologous recombination, does not require template homology^122^) or microhomology-mediated break-induced replication (MMBIR, repairing single-stranded DNA which shares microhomology with the 3’ end of the replication fork^123^). Another double-strand DNA repair mechanism, alternative end-joining, has been particularly implicated in translocations^122^.

Simple rearrangements usually arise from breakpoints restricted to one or two chromosomes^111,124^, and are common across many cancer types^21^. These events may be further sub-categorized according to whether they alter DNA copy number. Balanced variants, such as inversions and reciprocal translocations, result in relative conservation of copy number^111^, whereas unbalanced rearrangements (*e.g*. deletions, duplications, non-reciprocal translocations) lead to CNAs^111^. Rearrangements were first linked to oncogenesis with the identification of the *BCR*-*ABL1* gene fusion in leukemia^125^, and have since been implicated in the development of a number of cancer types^126–130^.

Two more complex processes related to simple rearrangements were identified recently: rigma events comprise cumulative clustered deletions occurring in *trans*. This process appears to be distinct from chromothripsis, in which clustered breakpoints are confined to one allele^110,115^. Pyrgo events were characterized by clustered duplications without a significant increase in copy number^115^. Relative to their simple counterparts, rigmas are enriched in late-replicating regions, fragile sites, and large genes. Similarly, pyrgo events occur more frequently in early-replicating regions and super-enhancers. These differences may be due to distinct selection pressures or driver processes^115^.

**Figure 3:**
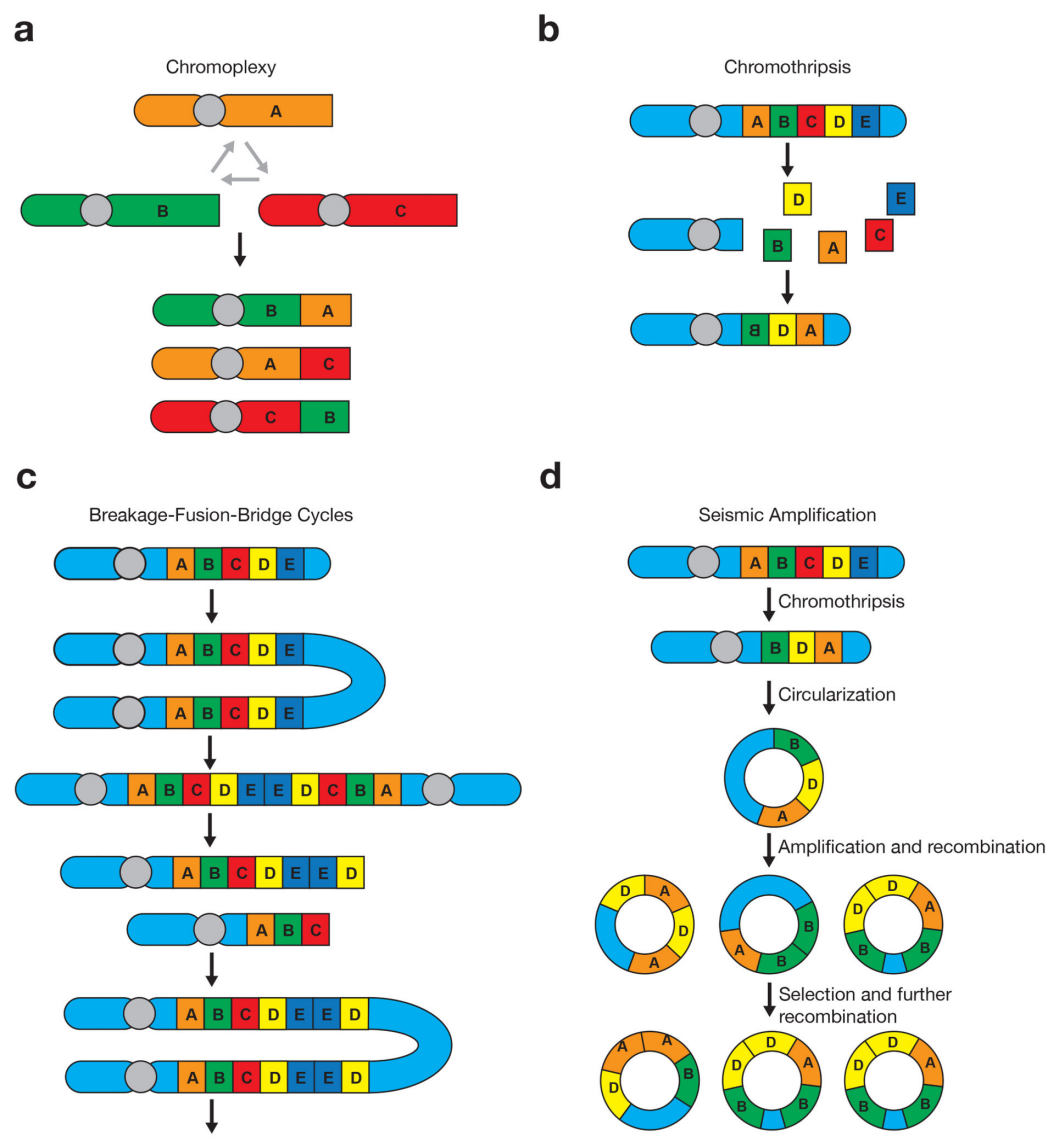
A summary of the processes underlying four classes of large-scale genomic rearrangements. **a**, Chromoplexy arises through translocations involving multiple chromosomes, generating apparent chains of rearrangements. **b**, In chromothripsis, chromosomes are shattered and near-randomly rejoined in a single event. **c**, Breakage-fusion-cycles are initiated by fusion of two sister chromatids, generating a dicentric chromosome. This chromosomal bridge breaks during the subsequent anaphase, resulting in double strand DNA breaks which may trigger further cycles. **d**, Seismic amplifications represent a combination of previously-described rearrangement events, whereby chromothripsis is followed by amplification through ecDNA formation.

Chromoplexy is characterized by interdependent, shuffled, apparent chains of rearrangements, typically involving multiple chromosomes^113,131^. These events are also likely related to simple rearrangements, namely balanced translocations, which can be viewed as chromoplexy chains involving only two chromosomes^21^ (Fig. 3a). As such, chromosome translocations and chromoplexy chains are likely the same entity. Breakpoints in chromoplexy may be associated with deletion bridges, or generate gene fusions with relative conservation of copy number^132^. These rearrangements underlie pathogenic gene fusions and enhancer hijacking events in a range of malignancies^21,109,116,119,131,133^, and may result in synchronous disruption of multiple oncogenes^113,132–134^. Chromoplexy is particularly prevalent in prostate cancer and frequently generates *TMPRSS2-ERG* driver fusions^113,116,134^. To date, chromoplexy events have been inferred solely from whole genome sequencing data, and the initiating mechanism is not currently well-characterized. However, chromoplexy chains are likely formed by double-stand breaks involving multiple chromosomes, followed by incorrect repair^111,135^. Chromoplexy chains often co-occur with other rearrangement patterns, such as chromothripsis (discussed below), and may have a causal role in some of these events^116,119,136,137^.

### Chromothripsis

Chromothripsis describes a phenomenon whereby chromosomes are shattered into fragments that are rejoined near-randomly in a single event^110^ (Fig. 3b). This generates massive, clustered rearrangements restricted to either a single chromosome, or localized regions of multiple chromosomes^110,138^. These events are prevalent across cancers and are particularly frequent in bone and soft tissue tumours^21,116,139^.

A set of criteria have been defined to distinguish chromothripsis from other rearrangement events^120^. First, as chromothripsis is a localized phenomenon, it leads to high density breakpoint clustering in a short genomic interval^110,120^. Moreover, these events are confined to one allele, and are frequently associated with loss of genomic segments^110,138^. Classical chromothripsis events in a diploid context should therefore show alternating segments with retention and loss of heterozygosity, leading to a characteristic profile of copy number oscillation between two states^120,139^. Finally, as rejoining of shattered chromosomes is a random process, breakpoints from the four classes of intrachromosomal rearrangements (tandem duplications, deletions, and head-to-head and tail-to-tail inversions) should be represented in approximately equal proportions^110^.

Chromothripsis events may occur in diverse genomic contexts, with localized rearrangements in otherwise quiet genomes, or within a background of more widespread genomic rearrangements^116,139^. The properties of chromosomal changes have been shown to differ widely across tumor types^116^, suggesting different underlying mechanisms or selective constraints. For example, in prostate cancer, classical single-chromosome chromothripsis is frequent, whereas in glioblastoma, small focal chromothripsis events are particularly common, and in melanoma, osteosarcoma and liposarcoma, chromothripsis events often involve multiple chromosomes^116^. Many of these events are followed by amplification, which we discuss in the following section.

From a mechanistic perspective, chromothripsis is one of the better-studied complex rearrangement processes. Polyploidy and abnormalities in *TP53* appear to predispose to chromothripsis in some tumor types, but it may also occur in diploid tumors with normal p53 function^121,139^. Chromothripsis can result from dicentric chromosome formation as part of breakage-fusion-bridge cycles (discussed below), or through DNA damage to lagging chromosomes in micronuclei^140^. This spatial restriction explains the clustered nature of the observed rearrangements^120,139^. Chromothripsis events arising through these routes show distinct genomic footprints and underlying DNA breakage mechanisms^141^. Events secondary to chromatin bridge breakage show highly localized breakpoints with frequent foldback inversions, whereas rearrangements following micronuclear rupture are distributed more evenly across chromosomes and show oscillation between two or three copy number states^141^. In chromothripsis resulting from chromosome bridge breakage, DNA breakage occurs by mechanical force^140^.

At mitosis, lagging chromosomes can recruit a nuclear envelope to form a micronucleus^142^. However, recruitment of key nuclear proteins to lagging chromosomes is inhibited by bundled spindle microtubules^143^, resulting in defective micronuclear function and collapse of the nuclear envelope during S phase^12–15^. On exposure to cytoplasm, DNA previously contained within the micronucleus is subject to damage and fragmentation^144^. This phenomenon seems to be a function of aberrant DNA repair: in experimental models, accumulation of DNA-RNA hybrids on chromatids within micronuclei ultimately results in induction of double strand DNA breaks, largely mediated through the base excision repair enzymes APE1 and MPG^144^. Experimentally induced loss of these proteins vastly reduces, but does not fully ablate, DNA damage accumulation in interphase^144^. These findings indicate a role for multiple processes in fragmentation of chromatids at chromothripsis, with both NHEJ and MMBIR representing possible additional candidates^120,121,145^.

DNA arrangements generated by a single chromothripsis event have the potential to yield multiple oncogenic alterations^120^. Consistent with this, the presence of chromothripsis confers a worse outcome in multiple cancer types^121,146–149^. Chromothripsis could promote tumorigenesis through focal deletion of tumor suppressor genes present on lagging chromosomes^38,46,110,116,139,150^. Rearrangements can also generate enhancer hijacking events and oncogenic gene fusions^119,151^. Finally, chromothripsis could result in oncogene amplification, for example through ligation of fragments to generate circular chromatids, leading to formation of ecDNA^110,114,139,145,150^ (discussed below). Chomothripsis-amplification has been reported in multiple tumor types, including sarcoma and glioblastoma^116,139^. In a recent pan-cancer study, chromothripsis followed by amplification of underlying oncogenes (likely through multiple independent physical processes) was the most prevalent consequence on cancer genes^116^, suggesting positive selection for such events, as discussed in the next section.

### Complex structural variants underlying high-level amplifications

High-level amplifications are frequent oncogenic events in cancer genomes and can be caused by multiple processes. First described in maize genomes^152^, breakage-fusion-bridge (BFB) cycles are now a recognized mechanism of gene amplification in multiple cancer types^153^. These rearrangements may be initiated by various events, including dysregulation or loss of telomeres^112,154^, fragile site expression^155,156^, and DNA breaks induced by replication stress^154^. The key event initiating BFB cycles is the fusion of two sister chromatids, generating a dicentric chromosome^157^. During the subsequent anaphase, this may break at any point between the two centromeres^154^, resulting in double-strand breaks which may in turn trigger further cycles (Fig. 3c). These events may be terminated by telomere stabilization, either through capping or by fusion with another chromosome^158^. Repeated cycles of chromosome breakage and end fusion result in a characteristic pattern of palindromic fold-back inversions and jumps in DNA copy number^112,154,158,159^.

Akin to mis-segregations, BFB cycles lead to uneven inheritance of genetic material. Over multiple iterations, this process may generate high copy-number heterogeneity, facilitating selection of cell populations with tumorigenic profiles. In human cancers, BFB cycles have been implicated in the amplification of specific oncogenes, including *ERBB2* in HER2-positive breast tumors^158–160^, *CCND1* in head and neck squamous cell carcinoma^161^ and *MET* in gastric cancer^162^. Tumors exhibiting the structural hallmarks of BFB cycles show shorter survival in patients with head and neck, and ovarian cancers^161,163^. Chromothripsis is often observed together with other rearrangement patterns^3^, such as BFBs^110,112,154^. Micronucleus formation may provide a unifying mechanism linking these two processes: broken bridge chromosomes have been observed to segregate into micronuclei, leading to chromothripsis^140^.

Recently, three further complex rearrangement processes generating gene amplification have been inferred from whole genome sequencing data. The first category, cycles of templated insertions, were described alongside a distinct computational approach for identifying and classifying complex rearrangements^21^. This process is characterized by duplication of multiple segments from different reference chromosomes with insertion into one derivative chromosome and appears to be an important cause of *TERT* activation in hepatocellular carcinoma^21^.

Tyfonas^115^ and seismic amplifications^114^ are complex amplification events that share underlying characteristics with ecDNA. Tyfonas generate elevated copy number and, similar to BFBs, are enriched in fold-back inversions^115^. However, these amplicons show higher copy number and typically contain a higher proportion of the genome than other amplification mechanisms^115,164^. Relative to ecDNA and BFBs, tyfonas were found to be enriched in sarcoma, melanoma and breast cancer^115^. These events are associated with the characteristic *MDM2*/*CDK4* amplification in dedifferentiated liposarcoma^115^, and may underlie the circular neochromosomes observed in this tumor type^115,165^.

Seismic amplifications involve chromothripsis followed by repeated circular recombination generating high-level amplifications on ecDNAs^114^ (Fig. 3d). ecDNA is often formed by the ligation of chromothriptic fragments, as ~30% of amplicons show evidence of chromothripsis^164^. Seismic amplifications appear to represent a combination of known mechanisms, but with distinct characteristics. For example, in contrast to tyfonas, fold-back inversions are lacking. These events may account for a proportion of previously unclassifiable rearrangements^21,114^. Other related rearrangement processes, such as BFB events, may also generate ecDNA^114,166^.

### ecDNAs

ecDNAs are double-stranded circular chromatids, ranging from approximately 100 kilobases to several megabases in size^167–170^. These elements are distinct from neochromosomes, lacking centromeric and telomeric sequences^164,165^. First described in neuroblastoma in 1965^171^, ecDNAs are now established as common carriers of amplified oncogenes^164^, known to promote tumorigenesis and mediate therapeutic resistance, and associated with worse outcome in multiple cancer types^164,168,172^. Although ecDNA in cancer has been recently reviewed elsewhere^168,173^, we outline some key results on ecDNA in tumor development.

ecDNA lacks centromeric sequences and therefore segregate unevenly at mitosis. This can lead to large increases in copy number, and expansion of intra-tumor heterogeneity over relatively few cell cycles, enabling rapid adaptation to environmental pressures^167,168,170,174–176^. The resulting genetically diverse pool facilitates selection of cells with mutational profiles conferring a fitness advantage^168,169^. Genes encoded on ecDNA show increased copy number, with concomitant elevation in mRNA transcript levels^168,174^. Interestingly, however, this increase in transcription is not solely a function of increased copy number: oncogenes amplified on ecDNA show higher levels of transcription than copy number-matched chromosomal DNA^164,167,168^.

ecDNA can also act as a mobile regulatory element^174^. Given that it exhibits reduced nucleosome compaction, with depletion of repressive histone marks and enrichment of active histone marks^167^, ecDNA shows significantly enhanced chromatin accessibility relative to linear chromosomes^164,167^. In addition, ecDNA shows widespread long-range chromatin connectivity, with contact sites predominantly in promoter regions^167^. These elements are enriched for enhancer signatures, and associate particularly with transcriptionally active chromosomal genes^167^. Given these observations, ecDNA is proposed to increase oncogene transcription by acting in *trans* as a mobile transcriptional super-enhancer^167,169^.

ecDNA also enhances oncogene transcription by hijacking regulatory elements in *trans*. DNA FISH of interphase nuclei revealed clustering of 10-100 ecDNAs^169^. Molecules in these hubs showed higher levels of transcription than those outside, allowing ecDNAs lacking an enhancer to access enhancer elements of nearby molecules^169^. This spatial localization is reported to depend on the bromodomain and extra-terminal domain protein BRD4^169^.

### Complex rearrangements and tumor evolution

As detailed above, complex rearrangement events may substantially alter copy number and expression of oncogenes and tumor suppressor genes, and generate oncogenic gene fusions. As such, they can be important tumor driver events^110,116^, generating so-called evolutionary hopeful monsters^177,178^ with key roles in tumor initiation, development and evolution. Multi-cellular organisms evolved multiple mechanisms to restrain the occurrence and/or fixation of these events, some of which might be unknown. For example, in the case of chromothripsis, an Aurora-B-mediated correction of kinetochore positioning avoids mis-segregation events^179^, and cGAS-STING-mediated clearance of micronuclei leads to autophagic degradation of DNA fragments^180^. Even after escaping the cell’s surveillance and clearance mechanisms, large-scale rearrangements would generally be expected to have a negative fitness effect, given the scale of their disruption. For example, chromosome shattering will break apart multiple genes and therefore, most chromothripsis occurrences would likely be lethal or lead to a selective disadvantage. The chromothripsis and other complex events observed in cancer genomes, hence likely represent a biased series of events, enriched for occurrences under positive selection.

As with CNAs, physical considerations also affect the frequency with which different complex rearrangements are observed. Larger chromosomes more frequently mis-segregate and become entrapped in micronuclei, where they may undergo chromothripsis, partially explaining the higher rates of chromothripsis observed in larger chromosomes^52^. Although chromosome size is predictive of chromosomal frequency of chromothripsis, correcting for chromosome size seems to reveal further trends related to their physical and selective constraints. Notably chromosome 17p has a significantly higher rate of involvement in chromothripsis events than expected from its size alone^72^. This could be due to both the selective advantage of disrupting *TP53* (located on 17p), and the relatively shorter telomeres of 17p^181–183^.

Analysis of cancer genomes provides insight into the timing of complex events in tumor evolution. Recent data suggest that chromothripsis is more commonly early and clonal rather than late and subclonal^110,116,121^, although there may be some exceptions^139^. Consistent with this, chromothripsis events are enriched in known cancer drivers and are particularly associated with driver gene amplification^3^. In acral melanomas, which frequently exhibit amplified chromothripsis, both chromothripsis and the subsequent amplification of the oncogene *CCND1* typically occur early in tumor evolution^116^. In lung adenocarcinomas, oncogene amplifications associated with complex rearrangements, including chromothripsis and chromoplexy, are also typically early events^119^. In contrast, although chromothripsis was found to be an early event in lung squamous cell carcinoma, when associated with *SOX2* amplification, it often appeared later in tumor development^116^. Although longitudinal sequencing showed that chromoplexy can occur both early and late in prostate cancers^184^, this generally appears to be a clonal event^116^. These observations are consistent with the known role of chromothripsis and chromoplexy in generating driver events or oncogenic gene fusions in multiple fusion-driven cancer types (including *EML4*-*ALK* in lung adenocarcinoma and *TMPRSS*2-*ERG* in prostate cancer)^21,109,113,119,131,133,134,137,185,186^, as well as having the potential to disrupt multiple drivers at once in a single catastrophic event. Indeed, in prostate and pancreatic cancers and acral melanomas, single complex events targeting multiple driver genes have been reported^113,116,187^.

Treatment-induced selective pressures may also result in complex rearrangement events occurring later in tumor evolution. Pre- and post-treatment sequencing of colorectal cancer biopsies revealed chromothripsis- or BFB-mediated amplifications of mutant *BRAF*, leading to resistance to vemurafenib^166^. ecDNA has been particularly implicated in acquired resistance to targeted therapy *in vitro*^175^. Glioblastoma cells harboring mutant *EGFR* on ecDNA reduce *EGFR* copy number in response to tyrosine kinase inhibitor administration, conferring drug resistance^175^. Similarly, extrachromosomal amplification of the *DHFR* and *BRAF* genes confers therapeutic resistance in cervical carcinoma and melanoma models, respectively^188,189^. In at least some cases, this process is dynamic. In glioblastoma, mutant *EGFR* copy number rebounds following drug withdrawal^175^. In melanoma, there is evidence of dynamic switching between ecDNA and chromosomal amplification in response to MAPK inhibitor administration^189^.

Data regarding when other complex rearrangements arise during tumor evolution are sparse. It is likely that *rigma* and BFB events occur early in the development of oesophageal^115^ and pancreatic^112^ adenocarcinoma, respectively. As BFB events are closely associated with chromothripsis^110,112,154^, this process may represent an early event in other tumors. However, direct evidence for the contribution and timing of complex events in tumor evolution is currently lacking^21,114,115^.

## Conclusion and future perspectives

Next-generation sequencing technologies have allowed the systematic and detailed identification of large-scale genome alterations in thousands of tumors. Numerous different types of complex rearrangements have been identified so far, as well as different CNA patterns. Their frequencies of occurrence result from the interplay between physical effects that determine their rate of formation, and the impact they have on cellular fitness. Both these factors are still poorly understood. A better understanding of the complex perturbations that CNAs and complex genomic rearrangements impose on cells will require the combination of experimental systems that can induce such alterations with more sophisticated computational models and analyses. By resolving these large-scale alterations at the single-cell level, we may be able to separate the influence of physical and selective factors, through a comparison of the rate of their occurrence in individual cells to the rate of their progenies’ expansion.

Large-scale genome alterations often play a crucial role in tumor initiation, development, and evolution. Timing inference from sequenced cancer genomes suggests that these complex events often are critical early driver events in tumor evolution, advocating a potential role in the initiation of some cancers. Some of these events, particularly those leading to the formation and further evolution of ecDNAs, are highly dynamic and can evolve quickly under selective pressures imposed by the tumor microenvironment or by cancer therapies. However, many unanswered questions remain. How do we more precisely time the occurrence of complex rearrangements in cancer development and evolution? Can we build statistical models that work from a single biopsy, or is longitudinal data necessary? What are the precise physical factors that determine their occurrence across cancers and cancer types? How do these changes affect phenotype, and how can we model or measure the fitness effects of complex events or large CNAs in a specific genomic context? How do we disentangle the underlying mutational processes, given that few large-scale CNAs and complex rearrangements are expected to be selectively neutral? How do we understand the interactions between such events?

Although CNAs and SVs are intimately linked, they are often considered separately, leading to limitations in their detection and characterization. Moreover, there is currently no consensus in the field on how complex SVs and their associated CNAs can be classified. A more integrated analysis of genomic rearrangements and CNAs is required to delineate their roles in tumor development. A CIN signature approach that includes both structural and CNA features^22,23^ would likely allow a better understanding of the processes that lead to these alterations. In addition, the detection and characterization of these events may be improved further with platforms that sequence longer reads, aiding the identification, precise reconstruction, and classification of complex rearrangement patterns.

## Data Availability

This manuscript contains no original research or analysis.
